# Synergistic Effects of Glass and Flax Fibers Reinforced in Fly Ash Geopolymer Matrix

**DOI:** 10.3390/ma19010102

**Published:** 2025-12-27

**Authors:** Kacper Oliwa, Semanur Efe, Beata Figiela, Kinga Korniejenko

**Affiliations:** 1 Faculty of Material Engineering and Physics, Cracow University of Technology, Jana Pawła II 37, 31-864 Cracow, Poland; kacper.oliwa@doktorant.pk.edu.pl; 2 CUT Doctoral School, Cracow University of Technology, Warszawska 24, 31-155 Cracow, Poland; 3 Faculty of Metallurgy and Materials Engineering, Manisa Celal Bayar University, Şehit Prof. Dr. İlhan Varank Yerleşkesi, Yunusemre, Manisa 45140, Turkey

**Keywords:** geopolymer, flex fiber, glass fiber, hybrid fibers

## Abstract

**Highlights:**

**What are the main findings?**

- This study compares fly-ash-based geopolymers reinforced with short glass fibers and flax fibers, as well as hybrid fiber reinforcement, which was not previously studied in the literature.
- Results show that 1 wt% glass fibers effectively enhance compressive performance and matrix densification.

**What are the implications of the main findings?**

- The fiber addition at the tested dosages does not improve flexural strength.
- Optimizing fiber content/dispersion and interfacial treatment is recommended.

**Abstract:**

This study compares fly-ash-based geopolymers reinforced with short glass fibers (GF) or flax fibers (FF). Four mixes were produced: reference (FA), 1 wt% GF, 1 wt% FF, and a hybrid (0.5 wt% GF + 0.5 wt% FF). These compositions were cast into prism and cube molds, cured at 75 °C for 24 h, and tested after 28 days. Mechanical testing included compressive strength and three-point bending, phase composition by XRD, and microstructure by optical and SEM microscopy. The GF composite showed the highest compressive strength (mean up to ~28–34 MPa versus ~17 MPa for the reference), while FF gave intermediate values (~11–22 MPa). During bending, the reference achieved the highest flexural strength (~5.5 MPa); fiber-reinforced mixes ranged from ~2.9 to 4.4 MPa. XRD indicated a typical amorphous aluminosilicate gel over crystalline remnants; SEM/optical observations revealed a denser, more compact matrix with fewer voids for GF systems, whereas FF and hybrid mixes exhibited localized porosity and fiber pull-out imprints affecting crack initiation/propagation. Overall, 1 wt% GF effectively enhances compressive performance and matrix densification, while fiber addition at the tested dosages does not improve flexural strength; optimizing fiber content/dispersion and interfacial treatment is recommended.

## 1. Introduction

Geopolymers are a class of inorganic polymers synthesized through the alkali activation of aluminosilicate-rich materials, such as metakaolin, fly ash, or slag [1,2]. Distinguished by their low carbon footprint, excellent chemical resistance, and good thermal stability [3,4], geopolymers have emerged as a promising alternative to conventional Portland cement-based materials [5,6]. Their synthesis involves the dissolution of aluminosilicate precursors in an alkaline solution, followed by polycondensation to form a rigid, three-dimensional network of Si-O-Al bonds [7]. The resulting matrix exhibits a ceramic-like structure at ambient or mildly elevated temperatures, making geopolymers attractive for construction, infrastructure, fire-resistant panels, and waste immobilization [7,8].

Despite their many advantages, geopolymers are brittle and have limited tensile and flexural strength. This mechanical limitation has prompted researchers to explore various reinforcement strategies [9]. In recent years, growing attention has indeed been given to the task of incorporating fiber reinforcements into geopolymers, and this is because high-performance sustainable construction materials are, in fact, in increasing demand these days. Fiber reinforcement improves the fracture toughness of geopolymers by bridging cracks, controlling crack propagation, and enhancing post-cracking behavior [10]. Among the various fibers investigated, glass fibers and natural fibers such as flax have both shown promising results, each offering distinct advantages [10,11].

Glass fibers are widely used in fiber-reinforced composites due to their high tensile strength, modulus, and relatively low cost [12,13]. They exhibit good compatibility with geopolymer matrices and can substantially enhance flexural strength and load-bearing capacity [14,15]. The incorporation of short, chopped glass fibers has been shown to reduce crack width and improve both strength and ductility [16,17]. Furthermore, the increasing emphasis on eco-friendly construction materials continues to accelerate the exploration of fiber-reinforced geopolymers as viable substitutes for conventional cementitious systems. Glass fibers are not only industrially scalable but also relatively low in cost, which makes them especially attractive for applications aligned with the principles of green construction and the circular economy [18].

In contrast, flax fibers, as a natural and renewable reinforcement, offer a sustainable alternative. Flax is widely available, lightweight, and exhibits good tensile strength, making it a suitable choice for eco-friendly composite development [19]. Natural fibers can contribute to energy absorption, improve impact resistance, and reduce the overall carbon footprint of the material [20,21]. However, they are more sensitive to moisture, alkalinity, and biological degradation, which can compromise long-term performance unless treated or modified [22]. Their bond with the geopolymer matrix also plays a critical role in determining the mechanical response of the composite.

The motivation behind comparing these two types of fiber reinforcement lies in their contrasting properties. Glass fibers offer high mechanical performance and durability, whereas flax fibers provide environmental benefits and lower density. By evaluating them separately in similar geopolymer matrices, it is possible to understand how each fiber influences the composite’s workability, flexural and compressive strength, and microstructure. Directly comparing glass and flax fiber reinforcements in fly-ash-based geopolymer matrices stems from their contrasting properties: glass fibers deliver superior mechanical performance and durability, while flax fibers offer environmental sustainability, renewability, and lower density. The scientific novelty of this work lies in the first direct hybridization and comparative evaluation of these fibers within the same geopolymer system, revealing how flax’s natural hydrophilicity challenges dispersion and interfacial bonding compared to glass, yet achieves comparable flexural enhancements when optimized. By systematically assessing flexural/compressive strength, mineralogical composition and microstructure, this study provides novel insights into fiber–matrix interactions specific to fly ash geopolymers. This paper presents a comparative study of geopolymer composites reinforced separately with glass and flax fibers. The aim is to assess the mechanical performance and structural behavior of each system, providing insight into the advantages, limitations, and potential application areas for both types of reinforcement. Such comparative analysis is essential to guide material selection in sustainable construction applications where both performance and ecological impact are critical considerations. From a practical standpoint, these results guide construction industry adoption of hybrid flax–glass systems, enabling lighter, sustainable panels with glass-like durability for non-structural elements like facades and partitions, while reducing embodied carbon versus traditional cement composites. This comparative analysis is essential for material selection in green building certifications, balancing performance requirements with ecological imperatives in real-world applications.

## 2. Materials and Methods

### 2.1. Materials

The main ingredient for making a geopolymer was fly ash (FA) from the coal-combustion power plant “Skawina” (located in Skawina, Lesser Poland, Poland). The physical composition of the fly ash was investigated in previous research [23]. These studies demonstrated that the fly ash particle size distribution features predominantly fine particles with uniform dispersion, favorably supporting the geopolymerization reaction. Specifically, the average values were D10 = 2.06 μm, D50 = 10.97 μm, and D90 = 25.06 μm [23].

**Figure 1:**
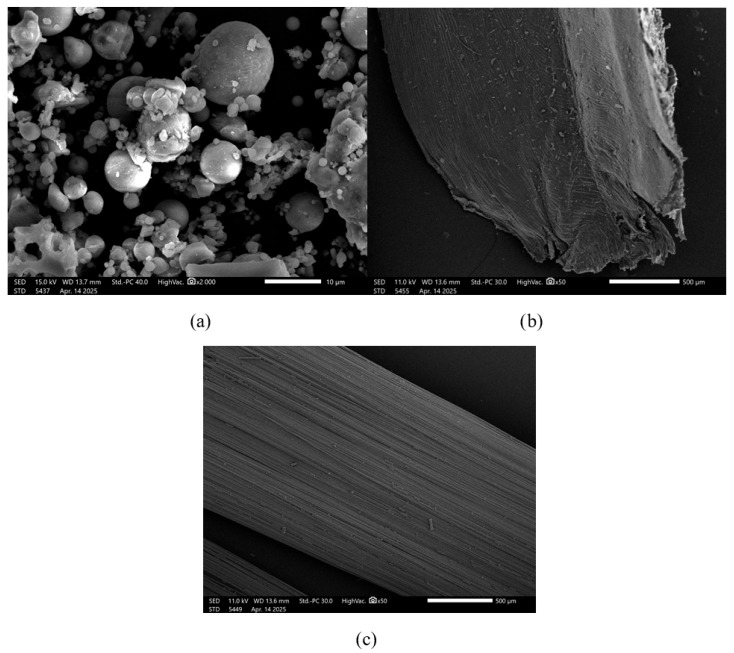
SEM images: (**a**) fly ash ×2000, (**b**) flax fiber ×50, and (**c**) glass fiber ×50.

The morphology of the fly ash (FA) is shown in Figure 1a. FA consists mostly of smooth, spherical particles with a regular shape. This uniform morphology enhances the workability of the mixture and lowers the demand for liquid components during geopolymer preparation [24]. This characteristic is especially advantageous when producing fiber-reinforced composites, which typically require a higher volume of liquid solution.

The morphology of the flax fiber (FF) is presented in Figure 1b. The image reveals a rough, irregular surface with noticeable longitudinal striations and a fibrous, layered texture. This coarse morphology can promote mechanical interlocking with the geopolymer matrix, enhancing fiber–matrix bonding. However, it may also result in localized stress concentrations and potential sites for crack initiation if not well impregnated with the binder.

Figure 1c shows the surface of the glass fiber (GF) at ×50 magnification. Unlike the flax fiber, the glass fiber displays a smooth and uniform surface, typical of synthetic reinforcements. This smoothness can reduce friction during processing and improve dispersion within the matrix, although it may also weaken interfacial adhesion unless surface treatments (e.g., silane coupling agents) are applied. The consistent diameter and defect-free surface of the glass fiber contribute to its predictable mechanical behavior in composite applications.

**Figure 2:**
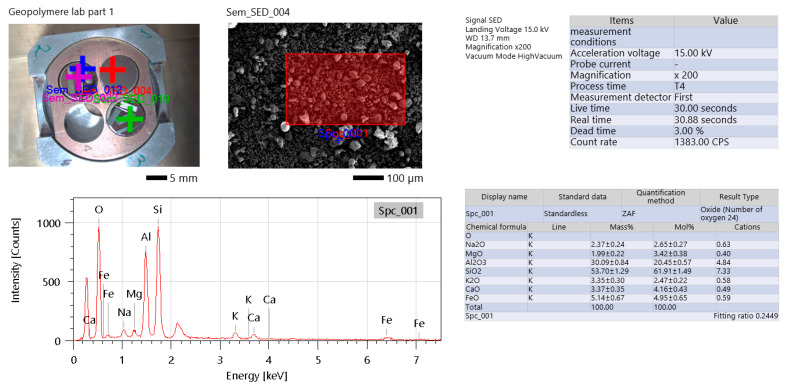
EDS analysis of fly ash (FA).

EDS analysis was performed on the raw materials. The provided analysis confirmed the oxide composition typical for fly ashes of class F [25]. The elemental composition of the fly ash confirms that it is based on silica and aluminum oxides (Al_2_O_3_—more than 30% and SiO_2_ almost 54%) (Figure 2). The elemental composition analysis revealed that iron (Fe) is an important component in the fly ash, suggesting the presence of hematite (Fe_2_O_3_) or other iron oxides [26,27]. Alongside iron, magnesium (Mg), potassium (K), and calcium (Ca) appear in variable amounts, most likely as mineral impurities such as dolomite or feldspars [26,28]. Sodium (Na) is present only in small amounts. These findings have important implications for geopolymer synthesis: high iron content can reduce reactivity in the geopolymerization process but may enhance thermal stability of the final product [29,30]. However, other research shows the positive influence of iron presence on the creation of geopolymer structure [29]. The presence of alkali metals like sodium and potassium is crucial for geopolymer network formation; however, their concentrations must be carefully balanced to prevent issues such as efflorescence [31]. It is also worth noting that the main sources of this element are activators such as water glass or sodium hydroxide, and the elements included in raw materials only supplement these kinds of elements [31].

Moreover, EDS analysis for fly ash was performed (Figure 2). Spectral data from Spc_001 confirm the presence of aluminosilicate bonds (AlSO_1-AlSO_4), which are essential for the development of the three-dimensional geopolymer matrix. The signal-to-noise ratio (SNR) in the spectra is moderate, indicating reliable, though not ultra-high-resolution, analytical quality. Measurement conditions during energy-dispersive spectroscopy (EDS) included a high accelerating voltage of 1500 kV, a standard setting for EDS, though such high energy can introduce beam penetration artifacts, especially in porous materials like fly ash. The count rate, measured at 1383 counts per second (CPS), falls within an acceptable range, although higher rates above 2000 CPS are generally preferred to improve data accuracy. Overloading method data show silicon (Si) as the dominant element, consistent with the aluminosilicate nature of fly ash. Minor variations in output percentages suggest that sample preparation was relatively homogeneous, although some localized heterogeneity cannot be ruled out. Notable limitations include a reference to “heavy steel SAMB”, indicating potential contamination either from the sample holder or preparation tools such as steel tweezers. Additionally, low-weight elements like carbon (C) and oxygen (O) are likely underdetected due to the inherent limitations of EDS in capturing light element signals [32].

Overall, the EDS data align with the expected composition of fly ash, reinforcing its viability as a precursor material for geopolymer synthesis. The presence of iron-rich phases was also confirmed by the X-ray diffraction (XRD) carried out in previous research [23].

**Figure 3:**
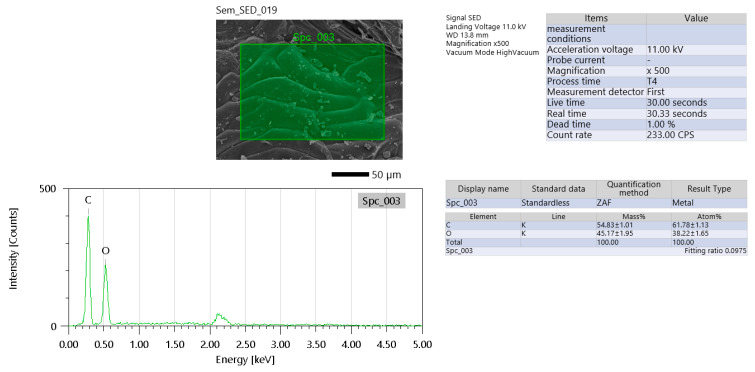
EDS analysis of flax fiber.

EDS analysis was also performed on the flax fibers (FFs) (Figure 3). The measurement conditions for this analysis employed an acceleration voltage of 11.00 kV, which is appropriate for detecting light elements such as carbon (C) and oxygen (O), although it may limit the detection of heavier elements. The count rate was recorded at 233 counts per second (CPS), which is relatively low and may suggest beam scattering from rough fiber surfaces or weak elemental signals due to the sample’s organic nature. However, the dead time was minimal at 1.00%, indicating that the detector functioned efficiently and without signal overload.

Elemental composition analysis revealed a high carbon content at 54.82 ± 1.01 wt% (61.78 ± 1.13 at%) and oxygen at 43.17 ± 1.95 wt% (58.22 ± 1.85 at%). These levels are consistent with the organic structure of flax fibers, primarily composed of cellulose and lignin [24]. Trace elements (less than 2%) were detected, likely originating from surface contaminants such as potassium (K) or calcium (Ca) introduced through environmental exposure or the fiber growth process.

Critically, the high carbon-to-oxygen ratio aligns with the typical chemical composition of cellulose (C_6_H_10_O_5_), which forms the primary structural component of flax [11,24]. Slight variations in this ratio can be attributed to the presence of lignin, which contains relatively more carbon, or pectin, which has a lower carbon-to-oxygen ratio. The absence of silicon (Si) and aluminum (Al) in the scan confirms that there was no interference from a geopolymer matrix, indicating the fiber sample was clean and free of mineral contamination in this particular measurement. If elements like K or Ca are observed in other scans, they would most likely be residual salts from fiber processing rather than inherent components.

Several limitations and potential artifacts must be considered. The low count rate could result from beam-induced damage to the organic fiber, causing charring, especially under higher acceleration voltages. Additionally, flax’s insulating nature may cause poor conductivity, and a thin or uneven gold coating might exacerbate this issue, leading to suboptimal signal detection. The absence of detectable sodium (Na) or magnesium (Mg), despite their known presence in natural flax, might be due to the overwhelming signals from carbon and oxygen masking these lighter elemental peaks [11].

For applications in geopolymer composites, these findings highlight several implications. The presence of hydrophilic oxygen-containing groups (such as hydroxyls) on flax fibers can lead to chemical interactions with the alkali components in geopolymer matrices, potentially resulting in degradation of the fibers over time. Therefore, pre-treatment methods such as silane coupling are recommended to enhance interfacial bonding and durability. Additionally, the high carbon content of the untreated fibers could interfere with the geopolymer crosslinking process, potentially weakening the overall composite structure if not properly managed [19,24].

**Figure 4:**
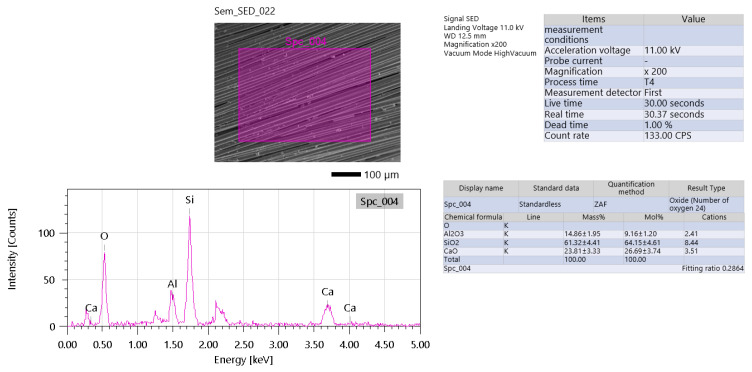
EDS analysis of glass fiber.

Also, as raw material, glass fibers were analyzed by EDS (Figure 4). The measurement conditions for this analysis utilized an acceleration voltage of 11.0 kV, which is optimal for detecting light to mid-range atomic number elements such as silicon (Si), calcium (Ca), and oxygen (O). The count rate was relatively low at 133 counts per second (CPS), indicating either sparse elemental signals or beam scattering, possibly due to the smoothness of the glass fiber (GF) surface. Despite the low count rate, the dead time was only 1.00%, demonstrating efficient detector performance without signal saturation.

Elemental composition data, expressed in oxide form, show that silicon dioxide (SiO_2_) is the dominant component at 61.33 ± 4.41 wt% (64.15 ± 4.51 at%), consistent with its role as a network former in typical E-glass compositions [14,33]. Calcium oxide (CaO) was also significantly present at 23.81 ± 3.33 wt% (26.69 ± 3.74 at%), functioning as a network modifier that enhances durability and improves resistance to chemical degradation. Aluminum oxide (Al_2_O_3_) was detected at a relatively low concentration of 1.43 ± 1.95 wt% (9.16 ± 2.0 at%), which is somewhat lower than expected for standard E-glass and could suggest a specialized glass formulation or a measurement anomaly. Trace elements—most likely magnesium oxide (MgO) or iron oxide (Fe_2_O_3_)—were also present in minor amounts, under 2% [14,33].

Key observations include the high SiO_2_ content, which aligns well with industrial standards for E-glass fibers, confirming the material’s identity. The presence of CaO is notable, as it indicates the use of a calcium-modified glass type, which is especially beneficial for applications in geopolymer composites due to its enhanced alkali resistance. The low Al_2_O_3_ content is atypical for standard E-glass and might reflect a specialty formulation or possible underestimation due to analytical limitations [14,33].

Some limitations and potential artifacts should be acknowledged. The low count rate could be attributed to inadequate conductive coating—since glass fiber is inherently insulating—or to beam deflection caused by the fiber’s smooth surface. Additionally, the absence of sodium (Na) and potassium (K) peaks is unusual for commercial glass fibers, possibly pointing to a low-alkali formulation or surface leaching effects that removed these elements from the fiber surface.

From a geopolymer composite perspective, this composition suggests several beneficial properties. The high SiO_2_/CaO ratio ensures good chemical compatibility with alkaline geopolymer matrices, reducing the risk of degradation. However, the smooth surface of the glass fibers may hinder interfacial bonding, which could be addressed by using silane coupling agents to improve adhesion. The presence of CaO further enhances long-term durability by mitigating the risk of alkali–silica reaction (ASR), making these fibers particularly suitable for reinforcement in geopolymer-based materials [26].

The alkali solution (NaOH 10 mol/dm^3^) was prepared by using a weighted amount of 400 g of technical flakes NaOH and adding 1000 mL of tap water. The solution was mixed, and then the aqueous sodium silicate was added in a ratio of 1:2.

Then, the alkaline solution was left to stand for 24 h.

Four types of samples were produced:

1. A reference sample.
2. A geopolymer containing 1% by weight of glass fibers (GFs).
3. A geopolymer with 1% by weight of flax fibers (FFs).
4. Hybrid geopolymer comprising 0.5% CF and 0.5% FF by weight.

The glass fibers were uniform and had a length of 3 mm. The FFs had a diversified shape and length between 2 and 5 mm.

The sample labels are listed in Table 1. Both types of fibers were used without any surface treatment.

A weighted amount of fly ash was put in the mixing bowl of the planetary mixer. Then, the alkaline solution was added in a liquid-to-solid ratio of 0.6 (l/s = 0.6). The ingredients were mixed for about 10 min for the basic mixture, and then the fibers were added to the paste and mixed for 5 more minutes to obtain a uniform distribution of fibers in the geopolymer matrix, in the case of fiber geopolymer types. The prepared paste was cast in the set of rectangular molds with dimensions of 50 mm × 50 mm × 200 mm, cubic molds of 50 mm × 50 mm, and plate mold of 200 mm × 200 mm. The molds were wrapped with foil. After that step, the molds were placed on a vibrating table to remove air bubbles. A set of molds was put into the laboratory dryer at 75 °C for 24 h. After that time, the samples were unmolded and cured for 28 days.

### 2.2. Methods

#### 2.2.1. X-Ray Diffraction

A diffractogram is the graphical output produced during X-ray diffraction (XRD) analysis. In this technique, the intensity of X-rays diffracted by a sample is measured and plotted as a function of the diffraction angle, commonly referred to as 2θ. This plot reveals crucial information about the material’s crystallographic structure.

Interpreting a diffractogram involves several key aspects. First, the overall appearance can help distinguish between crystalline and amorphous materials. Crystalline substances exhibit sharp, well-defined peaks due to their ordered atomic arrangements, while amorphous materials show broad, featureless humps, indicating a lack of long-range order.

Peak locations (2θ values) correspond to specific crystallographic planes within the material and are essential for identifying phases. These peaks can be matched with standard reference patterns, such as those in the Powder Diffraction File (PDF) database, for accurate phase determination.

The amplitude or intensity of each peak reflects the relative abundance of a phase or the preferred orientation of its crystallites. A higher peak implies a greater presence or alignment along a particular set of planes.

The number of peaks provides additional structural insight. A high number of peaks may indicate a multi-phase or polycrystalline material, resulting from varied nucleation and growth processes. Conversely, a limited number of sharp features typically suggests a single-phase or highly oriented crystal structure. If few or no sharp peaks are present, the sample is likely amorphous.

Finally, the full width at half maximum (FWHM) of each peak is an important indicator of crystallite size and internal strain. Narrow peaks suggest larger crystallites and lower microstrain, while broader peaks imply smaller particle sizes and higher internal stress. These parameters can be quantitatively assessed using the Scherrer equation, allowing estimation of crystallite dimensions and strain levels within the material.

#### 2.2.2. Compressive and Flexural Strength

Analysis of the compressive strength (CS) and flexural strength (FS) was performed to investigate how the carbon fibers and the natural fibers affected the mechanical properties of the geopolymers. The compressive strength was calculated using the following:

(1) $${\sigma_{c}=\frac{F_{c}}{A}}_{0}$$

where:

σc = Compressive strength (MPa) (standard symbol).

Fc = Maximum compressive load at failure (N).

A_0_ = Original cross-sectional area (mm^2^) (subscript “0” denotes initial condition).

Flexural strength is the material’s ability to resist deformation under load. In other words, flexural strength is a mechanical property that evaluates the stiffness of a material. For this test, the shape of the samples was a rectangular prism (cuboid), and the measurement of the length was performed by taking into consideration the a and b directions. The specimen was placed on two supports (distance between supports 150 mm), and a load F was applied at the center of it. Failure occurred when the strain or elongation exceeded the limit of the material.

The three-point bending test was conducted according to ASTM C78/C78M [34], with flexural strength calculated as follows:

(2) $$\sigma_{f=}\frac{3F_{f}L}{2bd^{2}}$$

where:

σf = Flexural strength (MPa) (standard symbol).

Ff = Peak load at fracture (N).

L = Support span (150 mm).

b = Specimen width (mm).

d= Specimen depth (mm).

#### 2.2.3. Optical and SEM Microscopy

Observation of the microstructure of geopolymer composites provides valuable insights into the adhesion between reinforcement fibers and the surrounding matrix, which can significantly influence the material’s mechanical properties. The microstructural analysis was carried out using a scanning electron microscope (SEM) and an optical microscope. To ensure electrical conductivity, the surface of the geopolymer samples was coated with a thin layer of gold. This method allows for high-resolution imaging and precise magnification. The investigation was conducted using a JSM-IT200 InTouchScope™ SEM (JEOL, Tokyo, Japan), and optical microscopy was conducted using Keyence VHX-X1 (Keyence International, Mechelen, Belgia).

## 3. Results and Discussion

### 3.1. X-Ray Diffraction

**Figure 5:**
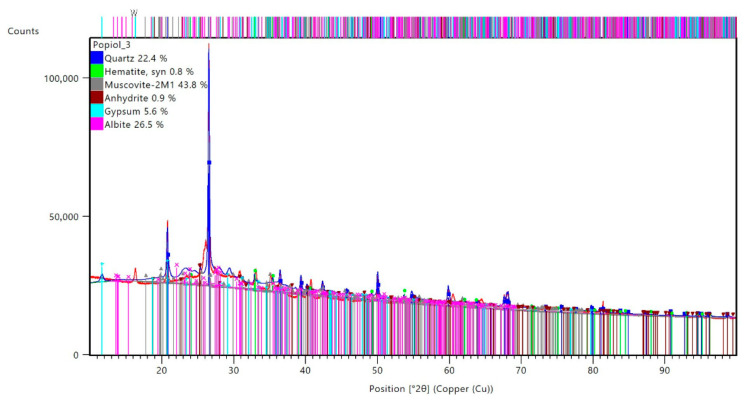
XRD diffractograms of fly-ash-based geopolymer.

The X-ray diffraction (XRD) diffractogram of the fly-ash-based geopolymer reveals a composite structure consisting of both crystalline and amorphous phases (Figure 5).

Dominant crystalline phases identified include muscovite-2M1 (43.8%), albite (26.5%), and quartz (22.4%), along with smaller quantities of gypsum (5.6%), anhydrite (0.9%), and hematite (0.8%). These phases are represented by sharp and well-defined peaks, indicating the presence of unreacted or partially reacted minerals originally present in the fly ash. The most intense peak corresponds to quartz, a common component in fly ash, known for its chemical inertness and contribution to structural stability.

Superimposed on these peaks is a broad amorphous hump between approximately 20° and 40° 2θ, which is characteristic of the disordered aluminosilicate gel phase formed during geopolymerization, such as sodium aluminosilicate hydrate (N-A-S-H) or calcium-substituted equivalents (C-A-S-H), depending on precursor chemistry. This amorphous phase is critical for the binding and cementing properties of geopolymers. The coexistence of crystalline and amorphous regions suggests that the geopolymerization process was only partially complete, a typical feature of systems cured at moderate temperatures or with low alkaline activation. Overall, the diffractogram confirms that the fly-ash-based geopolymer matrix consists of a complex blend of reactive gel and inert mineral phases, which together define the material’s mechanical and chemical performance. The identified phases confirm the geopolymeric characteristics of the analyzed materials [35,36].

### 3.2. Compressive and Flexural Strength

The necessary dimensions of the specimens were measured, and the tests were conducted. The results of the tests performed to determine compressive and flexural strength are presented in Table 2.

As shown in Table 2, samples without fiber reinforcement exhibited lower compressive strength values, reaching 16.94 MPa. The introduction of glass fibers contributed to a noticeable improvement in compressive strength—up to 28.70 MPa. The flax fibers do not significantly influence the values of compressive strength. In the case of flexural strength (Table 2), the highest values were recorded for the reference samples without fibers, with results of 5.49 MPa. The samples containing glass or flax fibers showed lower compressive strength. This indicates that in compressive strength tests, the addition of fibers did not lead to improved performance and, in some cases, may have slightly reduced the compressive strength of the composite—likely due to microcracks or the disruption of matrix homogeneity. The lowering of compressive strength in all cases was not an expected phenomenon, because most publications suggest that the flexural strength increases after the addition of fibers [24,37].

### 3.3. Optical and SEM Microscopy

**Figure 6:**
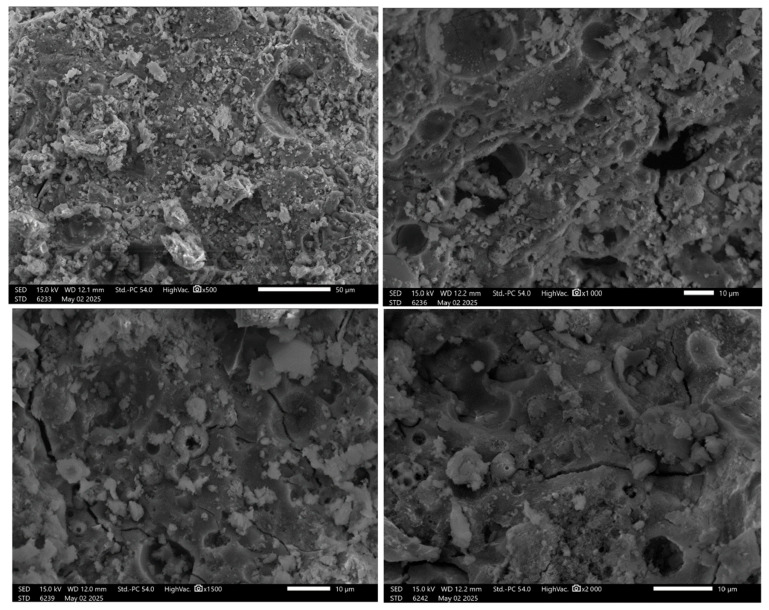
SEM images of the FA geopolymers.

The microstructural analysis of fly ash (FA) geopolymers under varying magnifications reveals the progressive changes in morphology associated with geopolymerization (Figure 6).

At ×500 magnification, the surface appears rough and uneven, with large, angular unreacted fly ash particles clearly visible. Some porous regions are also present, likely representing the initial or unactivated structure of the fly ash. Increasing the magnification to ×1000 provides a closer view, revealing more defined pores and reaction products. The structure at this stage appears more consolidated, with gel-like formations emerging; however, the presence of voids suggests incomplete geopolymerization or air entrapment.

At ×1500 magnification, the matrix appears more compact, with a noticeable reduction in unreacted particles compared to the earlier observations. A dense gel matrix is present, likely consisting of sodium aluminosilicate hydrate (N-A-S-H) gel, indicating successful geopolymerization. Another image at ×2000 magnification shows a slightly more porous structure, featuring mixed phases of partially reacted fly ash and geopolymer gel. Microcracks and pore clusters are evident in this sample, which may negatively impact mechanical strength.

In summary, these micrographs collectively illustrate the microstructural evolution of FA-based geopolymers. As the reaction progresses, whether through extended curing or enhanced activation conditions, the material transitions from a loose, porous matrix containing unreacted particles to a denser, more compact geopolymer gel phase. However, residual porosity and microcracks remain a concern, as they could influence the mechanical performance and long-term durability of the material. This kind of phenomenon was previously observed in the geopolymers’ composition with the addition of fibers [38,39].

**Figure 7:**
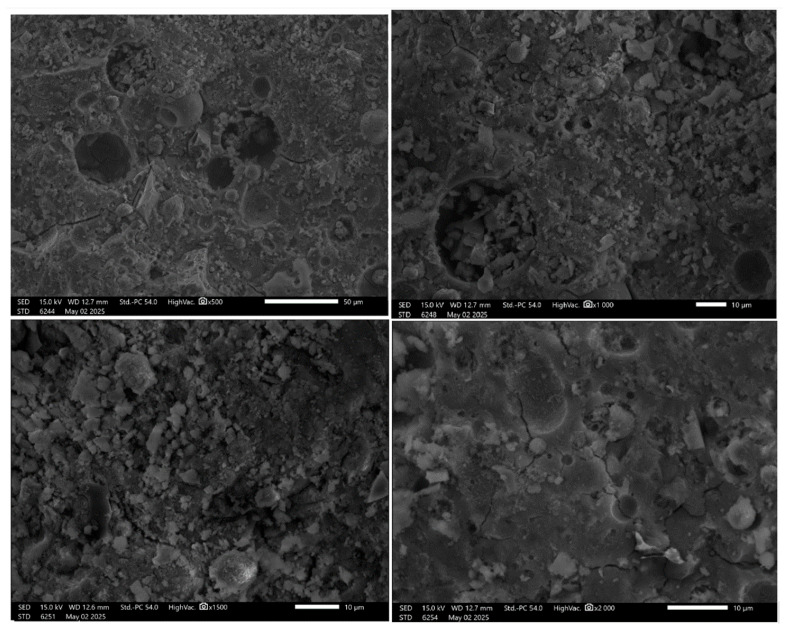
SEM images of the FA+FF+GF geopolymers.

The microstructural observations of FA+FF+GF (fly ash with flax and glass fibers) geopolymer samples across varying magnifications reveal key features related to fiber interaction, matrix development, and potential weaknesses (Figure 7).

At ×500 magnification, the matrix appears generally porous, characterized by large, rounded pores. These voids likely represent areas of fiber pull-out or regions where fibers were poorly bonded or removed, indicating zones of weak bonding or air entrapment, particularly around fiber interfaces.

At ×1000 magnification, a prominent circular cavity or fiber trace is visible, which may correspond to the location of a detached or poorly embedded glass fiber. The surrounding matrix appears more consolidated compared to fly-ash-only samples, although some unreacted or partially reacted particles remain present, suggesting incomplete geopolymerization in localized areas.

Under ×1500 magnification, the matrix shows increased density and reduced porosity. A compact, gel-like structure is apparent, consistent with successful geopolymerization. Angular particles within the matrix suggest the presence of residual unreacted fly ash or fragments of glass fiber, embedded within the hardened matrix. Such types of discontinuities were previously observed by other authors [2,24].

At the highest magnification of ×2000, the surface reveals a more complex morphology with interconnected voids. These voids may have formed as a result of fiber–matrix debonding, shrinkage-induced cracking, or internal stress development. Despite these defects, the matrix appears relatively homogeneous in areas free of voids or cracks

**Figure 8:**
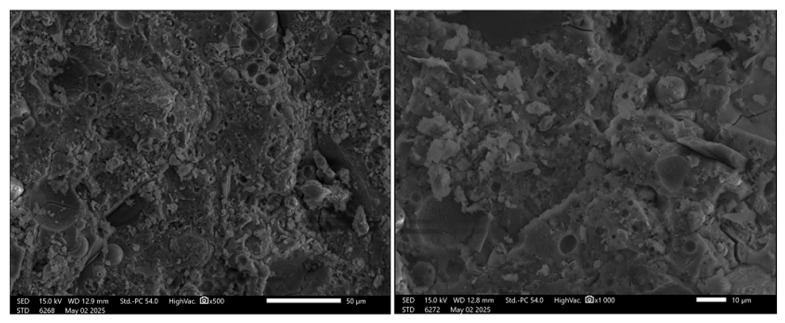
SEM images of the FA+FF geopolymers.

In comparison with the microstructure of fly-ash-only samples (Figure 8), the FA+FF+GF samples demonstrate more localized voids and cracking, which are likely associated with the presence and behavior of fibers within the matrix. However, these samples also show a more compact matrix in certain regions, indicating that fiber reinforcement may enhance the geopolymerization process. The observation of fiber pull-out or detachment is significant, as it highlights potential weaknesses in fiber–matrix bonding, which is critical for assessing the overall mechanical performance and integrity of fiber-reinforced geopolymer composites.

The SEM images of the FA+FF (fly ash with flax fiber) geopolymer matrix reveal a heterogeneous and porous microstructure, characterized by particles of varying sizes and morphologies (Figure 8).

Spherical particles of likely unreacted fly ash are distributed throughout the matrix, alongside gel-like binding phases that are indicative of geopolymerization products, such as sodium aluminosilicate hydrate (N-A-S-H) or calcium aluminosilicate hydrate (C-A-S-H) gels, depending on the material’s chemical composition.

Across all images, porosity and voids are clearly evident, with cracks and irregularly shaped pores suggesting incomplete geopolymerization or water evaporation during the curing process. These features become increasingly visible at higher magnifications, particularly in the bottom-right image, which exposes fine-scale porosity and potential microcracks. The interaction between particles is notably larger; particles appear embedded in a finer binder phase, with some showing signs of partial dissolution, supporting the occurrence of surface-level reactions between fly ash and the alkaline activating solution.

The images were taken at various magnifications, ranging from ×500 to ×2000, each offering unique insights into the material’s structure. The top-left image provides a broad view of the geopolymer surface, while the top-right and bottom-left images allow for more detailed observation of particle morphology and matrix texture. The bottom-right image highlights finer microstructural features, including pore distribution and microcrack formation.

**Figure 9:**
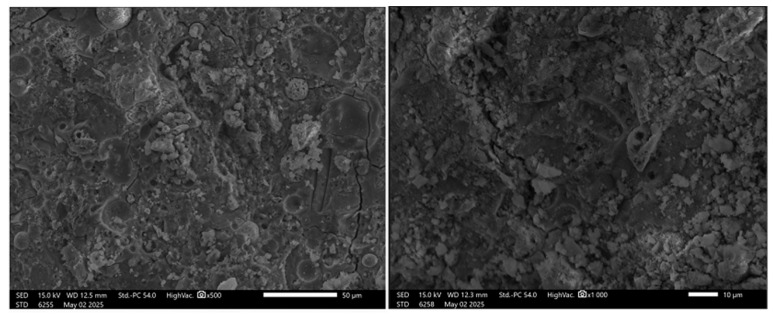
SEM images of the FA+GF geopolymers.

Overall, these SEM observations suggest that the FA+FF geopolymer matrix includes zones of incomplete reaction, with both unreacted and reacted particles coexisting within the structure (Figure 9). The presence of pores and cracks raises concerns about mechanical strength and long-term durability. Nevertheless, the general microstructural characteristics align with those typically observed in fly-ash-based geopolymers, particularly those cured at moderate temperatures, where complete densification is not always achieved. The other research suggests that the coherence between the FF and geopolymer matrix can be improved by proper fiber treatment [40,41].

The SEM analysis of the FA+GF (fly ash with glass fiber) geopolymer matrix reveals a noticeably more compact and denser microstructure compared to typical fly-ash-based geopolymers without fiber reinforcement. While glass fibers themselves are not distinctly visible in the images, potentially due to being embedded within the matrix or partially degraded, their presence is inferred from the more uniform microstructure and the apparent crack-bridging effects. These morphological characteristics suggest that the fibers contribute positively to the distribution of stress and the suppression of crack propagation.

**Figure 10:**
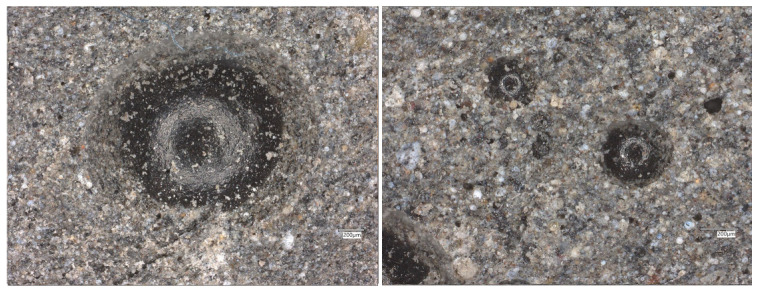
Optical images of the FA geopolymers.

The porosity in the FA+GF matrix appears to be significantly reduced when compared to FA+FF systems, such as those shown in Figure 10. There are fewer large voids, indicating improved particle packing and a more complete geopolymerization reaction. However, some small spherical pores or unreacted particles remain visible, consistent with partial reactions or trapped air. Despite this, the general reduction in voids points to a more effective reaction process and better structural cohesion.

In terms of cracking and interfacial behavior, only minimal microcracks or interfacial separations are observed across the images. This suggests that internal stress during curing has been better managed, likely due to the reinforcing role of glass fibers, which can help resist shrinkage and mitigate stress concentrations. The geopolymer gel phase presents as relatively homogeneous, indicating strong fiber–matrix bonding and effective integration of the glass fibers within the matrix. This is especially evident in higher magnification images (e.g., bottom-right at ×2000), which show fine grains embedded within a cohesive matrix—characteristics typical of a well-reacted and mechanically robust geopolymer. The images span several magnification levels: the top-left image at ×500 provides an overall view of surface density; the top-right at ×1000 highlights finer surface detail and compact regions; the bottom-left at ×1500, reveals smoother surfaces and evidence of particle bonding; and the bottom-right image at ×2000 offers insight into the detailed gel texture and possible fiber imprints or filler effects.

In conclusion, the FA+GF geopolymer matrix exhibits improved microstructural integrity compared to FA+FF composites. Despite the lack of clearly visible fibers, the enhanced matrix compaction, reduced porosity, and fewer intergranular defects strongly suggest that glass fiber reinforcement contributes to improved mechanical strength, crack resistance, and potentially greater durability. These features make FA+GF systems promising candidates for applications requiring high-performance, fiber-reinforced geopolymer materials.

The microstructure of the geopolymer synthesized solely from fly ash exhibits a relatively heterogeneous character (Figure 10).

Numerous spherical and semi-spherical particles can be observed within the matrix, corresponding to unreacted or partially dissolved fly ash cenospheres. The geopolymer gel surrounding these particles forms a continuous phase of varying brightness, indicating regions with different degrees of reaction. The darker areas correspond to unreacted aluminosilicate particles, while the lighter, more uniform regions represent the geopolymer gel (N-A-S-H type).

The presence of isolated pores and voids suggests local variations in reactivity and incomplete densification of the material. This morphology is typical for low-calcium fly ash geopolymers cured under moderate conditions and confirms the coexistence of amorphous gel and residual fly ash particles.

**Figure 11:**
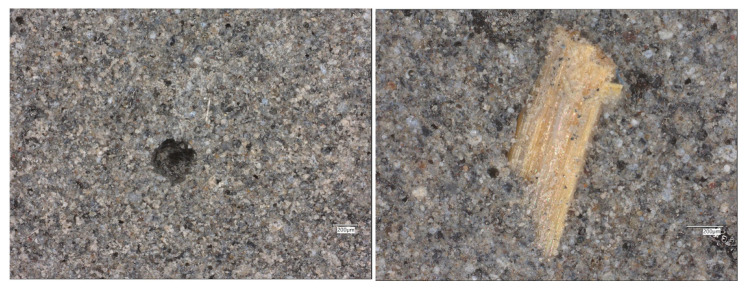
Optical images of the FA+FF+GF geopolymers.

The incorporation of both flax and glass fibers into the fly-ash-based matrix results in a more heterogeneous but compact microstructure (Figure 11).

The flax fibers appear as elongated, yellowish structures embedded within the grey geopolymer matrix. Their surfaces are well bonded to the surrounding gel, with no visible interfacial voids, indicating good adhesion and wetting during the geopolymerization process. The microstructure near the fibers shows localized densification of the gel phase, suggesting a positive interaction between the organic fibers and the inorganic binder. The presence of both natural and synthetic fibers enhances the microstructural integrity by bridging microcracks and reducing the effective porosity.

**Figure 12:**
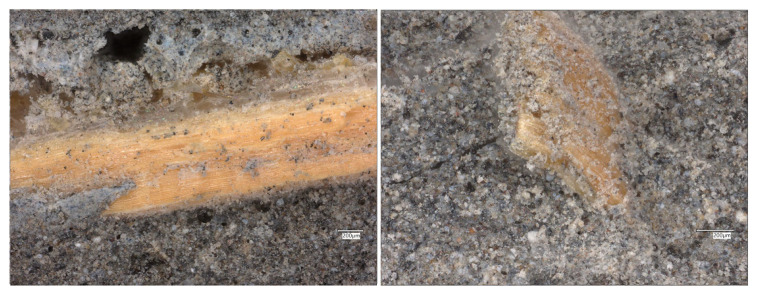
Optical images of the FA+FF geopolymers.

In the composite containing flax fibers, the optical images reveal elongated, well-preserved organic fibers distributed throughout the geopolymer matrix (Figure 12).

The fibers maintain structural continuity and exhibit good adhesion to the surrounding binder, indicating that the alkaline environment did not cause significant degradation. Occasional pores and voids are visible, especially near the fiber-rich regions, possibly resulting from trapped air during casting. Nevertheless, the overall structure is dense, and the flax fibers act as micro-reinforcement, limiting the development of microcracks and contributing to a more ductile fracture behavior of the composite.

**Figure 13:**
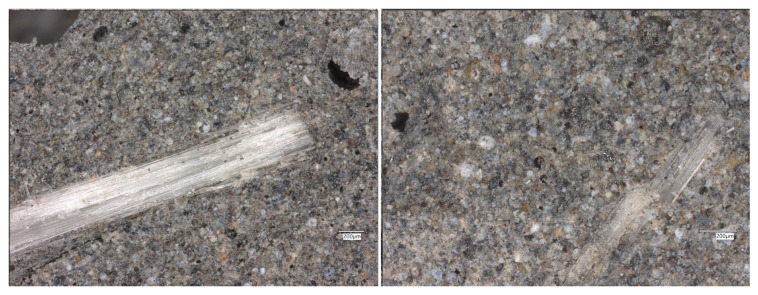
Optical images of the FA+GF geopolymers.

The microstructure of the geopolymer reinforced solely with glass fibers exhibits a uniform distribution of bright, elongated fiber fragments within the matrix (Figure 13).

The glass fibers show smooth surfaces and are well integrated into the geopolymer gel without signs of degradation or dissolution, confirming their chemical stability in the alkaline environment. The interfacial zones between the fibers and the matrix are compact, with no evident debonding or microcracks, suggesting strong adhesion. The surrounding matrix appears denser than in the reference FA sample, which can be attributed to the improved packing and reduced porosity resulting from the presence of fibers. The overall morphology indicates enhanced microstructural integrity and potentially improved mechanical performance.

However, while some of the literature reports fiber reinforcement enhancing both compressive and flexural strengths in geopolymers [4,42], our findings reveal a selective improvement in compressive strength without corresponding flexural gains. Similar results have been reported before [24]. This kind of behavior can be connected with improper fiber–matrix interfacial bonding and residual porosity visible in some microscopic images. Specifically, flax fibers primarily act as crack arrestors under compressive loads by redistributing stresses through the porous matrix, whereas in flexure, poor debonding and pull-out (due to hydrophilicity mismatch) fail to effectively bridge tensile cracks, limiting load transfer. This mechanistic decoupling underscores the need for targeted surface treatments to achieve balanced enhancements across loading modes.

## 4. Conclusions

This study evaluated the mechanical and microstructural properties of fly-ash-based geopolymer composites reinforced with flax and glass fibers.

The results of the flexural strength tests demonstrated that the addition of fibers did not lead to a significant improvement in flexural performance. In fact, samples without fiber reinforcement (reference samples) showed slightly higher compressive strength than those with added flax or glass fibers. This may be due to the introduction of microcracks or imperfections caused by uneven fiber distribution or disruption of matrix homogeneity.

In contrast, the compressive strength tests revealed a substantial enhancement in performance with the inclusion of fibers. Particularly, glass fibers significantly improved compressive strength, with some samples achieving values over 30 MPa. Flax-fiber-reinforced composites also exhibited better flexural performance than the reference samples, although to a slightly lesser extent than glass fiber counterparts. These findings confirm that fiber addition is more beneficial in enhancing flexural behavior rather than compressive resistance.

SEM and optical microscopy analyses further supported the mechanical results by showing a good bond between the fibers and the geopolymer matrix. The structure of the composites appeared compact, with minimal voids, and both flax and glass fibers were effectively integrated into the matrix. This likely contributed to improved stress transfer and fracture resistance under bending loads.

In conclusion, glass-fiber-reinforced geopolymer composites demonstrated superior mechanical behavior, particularly in flexural strength, making them suitable candidates for applications requiring high tensile or bending resistance. Flax fiber composites, while environmentally favorable, also improved performance compared to the baseline material, highlighting their potential for sustainable construction.

Based on the experimental results demonstrating suboptimal fiber dispersion and weaker interfacial bonding in flax-reinforced geopolymer composites, the authors propose several targeted optimization strategies for future research. Future studies may focus on optimizing fiber content and improving fiber dispersion to further enhance the mechanical performance of geopolymer composites. This can be accomplished by experimenting with different additives, including superplasticizers. This work could also focus on the development of effective methods of chemical treatments of flax fibers (e.g., silane or alkali pre-treatment) to enhance surface compatibility with the matrix. To enhance the environmental benefits, attention should be placed on sustainable flax variants through the use waste fibers combined with enzymatic treatments. After this step, the research should focus on functional properties, including thermal conductivity, acoustic absorption, and long-term durability through accelerated aging protocols (e.g., water absorption and freeze–thaw cycles). These evaluations will provide critical data on sustainability and service life, complementing the mechanical enhancements already identified.

## Figures and Tables

**Table 1:** Samples composition.

Type of Geopolymer	Fly Ash [g]	Alkaline Solution [g]	Glass Fiber [g]	Flax Fiber [g]
1.	3000	1800	-	-
2.	3500	1800	35	-
3.	3500	1800	-	35
4.	3500	1800	17.5	17.5

**Table 2:** Compressive and flexural strength of geopolymer samples.

No	Geopolymer Type	Compressive Strength	Flexural Strength	ρ (g/cm^3^)
1	Fly Ash	16.94	5.49	1.62
2	Fly Ash–Flax Fiber–Glass Fiber	15.56	3.00	1.51
3	Fly Ash–Flax Fiber	15.45	3.57	1.48
4	Fly Ash–Glass Fiber	28.70	3.45	1.58

## Data Availability

The original contributions presented in this study are included in the article. Further inquiries can be directed to the corresponding author.
